# Women with gestational diabetes in Vietnam: a qualitative study to determine attitudes and health behaviours

**DOI:** 10.1186/1471-2393-12-81

**Published:** 2012-08-09

**Authors:** Jane E Hirst, Thach Son. Tran, My An T Do, Forsyth Rowena, Jonathan M Morris, Heather E Jeffery

**Affiliations:** 1 Department of Obstetrics & Gynaecology, Sydney Medical School- Northern, University of Sydney, Royal North Shore Hospital, Sydney, NSW, Australia; 2 Hung Vuong Hospital, 128 Hong Bang St, District 5, Ho Chi Minh City, Viet Nam; 3 Perinatal Research Group, Kolling Institute, University of Sydney, Sydney, Australia; 4 International Women and Children’s Health, Sydney School of Public Health, University of Sydney, Sydney, Australia

**Keywords:** Gestational diabetes, Women, Vietnam, Developing countries, Focus group, Qualitative research, Pregnancy complications, Health promotion, Diabetes education

## Abstract

**Background:**

Diabetes is increasing in prevalence globally, notably amongst populations from low- and middle- income countries. Gestational Diabetes Mellitus(GDM), a precursor for type 2 diabetes, is increasing in line with this trend. Few studies have considered the personal and social effects of GDM on women living in low and middle-income countries. The aim of this study was determine attitudes and health behaviours of pregnant women with GDM in Vietnam.

**Methods:**

This was a qualitative study using focus group methodology conducted in Ho Chi Minh City. Pregnant women, aged over 18 years, with GDM were eligible to participate. Women were purposely sampled to obtain a range of gestational ages and severity of disease. They were invited to attend a 1-hour focus group. Questions were semi structured around six themes. Focus groups were recorded, transcribed, translated and cross-referenced. Non-verbal and group interactions were recorded. Thematic analysis was performed using a theoretical framework approach.

**Results:**

From December 2010 to February 2011, four focus groups were conducted involving 34 women. Median age was 31.5 years (range 23 to 44), median BMI 21.8 kg/m^2^. Women felt confusion, anxiety and guilt about GDM. Many perceived their baby to be at increased risk of death. Advice to reduce dietary starch was confusing. Women reported being ‘hungry’ or ‘starving’ most of the time, unaware of appropriate food substitutions. They were concerned about transmission of GDM through breast milk. Several women planned not to breastfeed. All felt they needed more information. Current sources of information included friends, magazines, a health phone line or the Internet. Women felt small group sessions and information leaflets could benefit them.

**Conclusions:**

This study highlights the need for culturally appropriate clinical education and health promotion activities for women with GDM in Vietnam.

## Background

The global increase in the prevalence of diabetes is occurring mostly in low and middle-income countries. It is estimated that 143 million women were living with diabetes in 2010 predicted to rise to 222 million by 2030 [1]. Gestational diabetes mellitus (GDM), defined as the new diagnosis of glucose intolerance in pregnancy [2], develops in around 1 in 25 pregnancies. In China the prevalence of GDM is rising [3], with similar trends noted in India [4] and other low-income countries. Type 2 diabetes has been demonstrated to be increasing in Asia [5], and the prevalence of GDM generally parallels type 2 diabetes in different populations [6]. The International Diabetes Federation recognise identifying and treating GDM as a global priority [7].

During pregnancy, GDM places the newborn at risk of macrosomia, birth trauma, stillbirth, prematurity, respiratory distress syndrome, neonatal hypoglycaemia and jaundice [8]. Tight blood glucose regulation largely ameliorates the adverse outcomes associated with GDM [8-10], although evidence on how to achieve this in low resource settings is limited. Whilst GDM usually resolves after delivery, these women have up to a 50% risk of developing type 2 diabetes within 10 years [11]. Thus the identification of GDM offers a chance to improve pregnancy outcomes and identify women to target dietary and lifestyle health promotion messages.

Our previous study demonstrated that in women undergoing routine antenatal care in Ho Chi Minh City, Vietnam, the prevalence of gestational diabetes is 6%, using the American Diabetes Association 2010 criteria [12]. This rises to 20% if the proposed universal International Diabetes in Pregnancy Study group criteria [13] were applied [14].

The majority of research on gestational diabetes has focused on epidemiological, pathological and biological aspects of the condition. Few authors have considered the social and behavioural effects of the diagnosis from the viewpoint of the women affected [15]. In line with the Health Belief Model, a woman’s perception of the threat of GDM to herself and her baby, and her belief in her own capacity to moderate the course of the disease can influence compliance with therapy [16]. Studies in low socioeconomic and migrant groups in North America [17] and Australia [18] show that the diagnosis of GDM can be associated with anxiety and confusion. Financial, language and cultural barriers can influence capacity to comply with health care advice.

In Vietnam, the experiences of women who are diagnosed with GDM and the challenges they experience in managing this condition have not previously been studied. The main aim of this study was to investigate these factors in order to determine what information and support is most needed to improve perinatal outcomes.

## Methods

This qualitative study used focus group methodology. This method was selected as the group interaction can encourage the generation of ideas and the expression of shared experiences and common viewpoints [19].

There were four focus groups conducted with between 6-10 women in each (total of 34 women). After three groups it was felt that data saturation had been reached. Another group was conducted to validate this finding. Women gave written consent and received information describing the purpose of the research. The groups were conducted in a quiet room in the hospital away from other clinical activities. The same facilitator led all focus groups. Focus groups were digitally recorded, transcribed and translated from Vietnamese into English by two independent translators with medical experience and then cross-referenced. Field notes and observations of group interactions were added to the transcript by the facilitator. Each session lasted between 60-90 minutes.

Group discussions were themed around six pre specified areas representing key aspects of pregnancy and diabetes care delivery in this setting. Open ended questions were structured around: understanding of the diagnosis of GDM, beliefs about the effects of GDM on their pregnancy, dietary changes, monitoring blood glucose levels, intention for breast feeding and how they would like to receive more information about GDM.

### Study subjects

This study was conducted at the Hung Vuong Hospital, Ho Chi Minh City, Vietnam. This hospital is a referral and local maternity facility for women residing in Ho Chi Minh City and the surrounding urban provinces. In 2010 the hospital conducted around 35 000 deliveries. In 2010 a policy of universal screening for GDM was introduced. This involved all pregnant women who were not known to have type 1 or 2 diabetes being offered a 75g 2-hour fasting oral glucose tolerance test at 28 weeks gestation. Women who were positive according to the 2010 American Diabetes Association criteria for GDM [12] were notified that they had GDM and were referred for management in the high-risk antenatal clinic. There they received dietary advice from a physician and were recommended to initiate blood glucose monitoring, either self-monitoring at home or weekly or bi weekly monitoring as an outpatient in the hospital. Women with persistently raised fasting (>126mg/dL, 7.0 mmol/L) or 1-hourpostprandial glucose (>200mg/dL, 11.1 mmol/L) were commenced on insulin. The hospital lacks formally trained nutritionists, endocrinologists and diabetes educators.

Women that were aged over 18 years and who had been diagnosed with GDM were eligible to participate. These women were purposively sampled to obtain a targeted representation of different severities of disease and gestational ages (28 weeks to 38 weeks). Women were very interested in participating in this study, with only one woman approached not agreeing to participate. Prior to the focus group, one of three trained research midwives obtained written consent and basic data on history and risk factors for GDM (Table1). Participation was voluntary and not remunerated. As an incentive to participate, women were informed that they would have an opportunity to ask a doctor with an interest in nutrition and a senior obstetrician (TST) questions around gestational diabetes after the session. Women were not given any additional resources about GDM nor additional counselling from a health professional prior to the focus group session compared to other women undergoing standard care for GDM in this setting.

**Table 1:** Maternal characteristics

**Characteristics**	**Group 1 (n = 10)**	**Group 2 (n = 13)**	**Group 3 (n = 6)**	**Group 4 (n = 6)**	**Total** **(N = 35)**
Age*	30.5 (23-41)	32 (28-38)	35 (27-40)	30 (27-44)	32 (23-44)
BMI*	22.4	20.7	22.0	19.1	21.7
	(18.3-28.8)	(17.3-31.3)	(18.0-27.3)	(17.3-23.0)	(17.3-31.3)
Ethnicity (Vietnamese)	10	12	6	6	34
Nulliparity	5	8	2	3	18
Highest Educational Level					
Primary	2	0	0	2	4
Secondary	8	11	4	2	25
Tertiary	0	2	2	2	6
Past Obstetric history	0	0	0	0	0
GDM	0	1	1	1	3
Stillbirth Macrosomia (> 3.8kg)	3	1	2	1	7
Family history of DM	0	5	3	0	8
Current treatment					
Insulin	2	4	1	3	10
Dietary modification	8	9	5	3	25

*median (min, max).

### Analysis

The focus group transcripts and field notes formed the raw data for analysis. A theoretical framework approach was used to analyse the transcripts using Nvivo 9 (QSR International). Using this approach the analysis is driven by predetermined research questions or themes, which provide a framework to group and then synthesise information, which is then used to develop an explanatory model [19]. Within each theme, categories were developed (Table2), with consideration of divergent views. After a preliminary explanatory model was reached, the categories were read again within the context of the original transcripts (re-contextualisation) and discussed with the facilitator to ensure the interpretation was true to the intended meaning of the participants. Direct quotations of opinions that summarised major themes or captured the attitudes of several participants have been given. These are reproduced without correction of grammatical errors to ensure maximum retention of meanings. The interpretation of subtle meanings and strength of the adjectives used to describe women’s attitudes could have been misinterpreted in the translation process. In an attempt to minimise this, the facilitator (who is fluent in Vietnamese and English), read over the transcripts to ensure the intended meanings were translated with fidelity. 

**Table 2:** Themes and subcategories from focus group analysis

**Theme**	**Main categories from focus groups**
Diagnosis and aetiology of GDM	·Confusion, anxiety and concern
	·Possibly due to genetic/hereditary factors
	·Toxic chemicals in food
	·Eating sweet foods in pregnancy.
Effects of GDM on pregnancy	·Fear of preterm birth, growth restriction, stillbirth
	·Uncertainty about safest mode of delivery
	·Mixed knowledge about the future risk of type 2 DM and other effects on the mother.
Dietary changes	·Confusion about exactly what to do
	·Feelings of hunger and starvation
	·Difficulties in replacing rice
	·Confusion whether to reduce portion size or total amount consumed
	·Specific food groups being prohibited or permitted (milk, fruit, red meat)
Blood glucose monitoring	·Lack of awareness of meter loan scheme
	·Fear of blood loss/pain from regular finger prick testing
	·Uncertainty about interpreting results
	·Inconvenience of coming to the hospital for testing
Breast feeding	·Fear of transmission of GDM to the baby
	·Preference for breast feeding for immune benefits
	·Supplementation with formula
	·Will take the advice of the doctor
Sources of information	·Sources of information from friends, family, other patients, Internet, magazines, health info phone service.
	·Desire for more information, particularly about diet and explaining effects of GDM on pregnancy.
	·Public clinic doctors very time pressured and unable to have time to answer all questions
	·More detailed leaflets needed with specific dietary advice
	·Group sessions with a senior clinician

### Ethical approval

Ethical approval was obtained from the University of Sydney (HREC approval number 13200) and the Hung Vuong Hospital Ethics Approval Board (approval number 725/QĐ-BVHV) prior to commencement of the study. All participants gave informed consent and were made aware that they could leave the group at any time and that data would be de-identified for the purpose of analysis and dissemination.

## Results

### Confusion and concern about the diagnosis of GDM

Prior to undergoing testing for GDM, women stated they had understood little about the reasons for the testing. Once diagnosed with GDM the most common expressed feeling they stated they felt was anxiety. Several participants used the (translated) words ‘worry’ and ‘fear’ to describe their feelings at the time of diagnosis. There was confusion about the aetiology of GDM.Women gave different explanations about why they had acquired GDM, ranging from a family predisposition to modern dietary habits. Most women were unsure of the aetiology. Several women blamed themselves for not controlling their diet.

"“*My doctor did not explain much about this condition. She only noted this condition was resulted partly from gene and partly the food we consume nowadays. Our food contains many kinds of chemicals that may cause diabetes. I could not understand the disease”*"

"*“I was so hungry I kept eating as usual. That must the reason I’m here today”*"

### Fear of adverse effects from GDM

Women were very concerned about the potential adverse effects of GDM on their baby. The major concerns were premature delivery, growth restriction and stillbirth. Common themes expressed were:

"*“It may slow baby’s growth, sudden death…babies easily have low blood sugar levels or more likely be born with abnormalities”*"

"*“..my baby might die if I’m not on diet”*"

Several women were uncertain about the risks that GDM posed for themselves during the pregnancy and after the birth, with other unrelated symptoms being attributed to the GDM.

"*“Some nights I felt it hard to breathe, I guess it’s resulted from high sugar in my blood”*"

Some women were aware of the increased risk of type 2 diabetes after the pregnancy, although many women attributed this to luck. Knowledge around need for testing for type 2 diabetes after the pregnancy was low. Women felt that testing would likely be necessary but were not aware when this should occur.

### Dietary changes “I think it’s so tough for us”

The major area of concern and the topic around which most discussion occurred was around changes in diet. Women reported fear of high blood glucose levels (BGL). There was confusion over exactly what dietary changes were recommended, with exclusion of food groups and drastic restrictions of intake reported by some.

"*“I knew it (consuming rice) was wrong, but I didn’t have any means to quantify”*"

"*“I am not sure which food should be taken and which should not be”*"

The solution to maintaining low BGL for many women was to restrict their dietary intake, resulting in feelings of hunger.

"*“I always feel starved but I dare not eat”*"

"“*I was asked to take only half a bowl each meal, but I am so hungry I eat as usual”*"

Participants in all groups reported feelings of hunger or starvation. Most women felt that they had to bear this for the sake of the baby, although there were several cases of women reporting their babies were growth restricted. Women felt that they had to trust their doctor, yet they were concerned about the effects of the recommendations.

"*“I’m afraid this diet won’t provide enough nutrients for the baby, but the doctor told me to do that”*"

"*“…I was too tired to be hungry, or even thinking about eating. As a result I could not gain weight and the baby became malnutritious (sic)”*"

The advice that most women reported being given was to ‘reduce starch’ intake in their diet. For Vietnamese women this advice was understood as reducing rice, the dietary staple in Vietnam.

"“*We Vietnamese are used to eating rice. We are thus not comfortable to have alternatives to rice.”*"

With a lack of counselling and advice about appropriate food substitutions, women experimented with alternate food sources.

"*“ (I thought) sticky rice could make me full longer, I thus took it. My blood sugar then rose up to 200 (mg/dL, 11.1mmol/L) and I had to admit to hospital”*"

"*“I change my food everyday to see which one is best for me”*"

Another food repeatedly mentioned in the groups was milk, with sweetened milk and pregnancy formula for mothers being very popular in Vietnam. Several women commented they had changed to sugar free milk, although were concerned about the nutrient value of this.

"*“I had to drink milk because I felt hungry, and I want to feed my baby”*"

"*“No sugar milk seems not to make me full. Rice makes me full, but starch is prohibited. It’s really hard for us.”*"

### Blood glucose monitoring: access and understanding barriers

Women expressed concern about blood glucose monitoring, with the majority being uncertain of the rationale behind testing and the interpretation of results. Some expressed concern about the effects of blood loss from repeated tests.

"*“I guess … I would be fatigued because of blood loss if I keep getting blood for checking every day until delivery”*"

Monitoring of blood glucose amongst women who were out patients ranged from 4 times a day using a personal meter, to twice weekly, weekly or second weekly monitoring in the high-risk pregnancy unit. For women with unstable diabetes admitted to the hospital, monitoring occurred up to 6 times per day. The hospital has a scheme whereby women can have a loan of a glucose meter free of charge. Several of the women in the focus groups were unaware of this scheme. Others did not feel confident in performing and interpreting the tests themselves. Thus most women preferred to come to the hospital for BGL monitoring. Several women stated this was inconvenient, however they felt they must do this for the wellbeing of the baby.

"*“Of course it is time consuming and affects my work a lot.”*"

Weekly blood glucose monitoring was not limited to women with mild diet controlled GDM. One woman reported being commenced on insulin and waiting 7 days before being asked to come back to the hospital for a BGL check. At that time her BGL was 8.8mmol/L fasting (recommended < 5.1 mmol/L).

One woman had had two previous pregnancies, the first resulting in a stillbirth and the second a macrosomic 5.1kg infant. These are both conditions associated with poorly controlled GDM. She stated that this pregnancy she preferred to have blood glucose monitoring once a week, as she disliked needles.

Information on what the optimal blood glucose ranges for fasting, pre- and post-prandial should be was also unclear to most of the participants. One woman stated that she knew the normal range by reading the leaflet in the blood glucose meter pack, a range likely to have been written for type 2 diabetes.

### Breast-feeding: balancing conflicting information about benefits for baby

There was a wide variation in opinions amongst women with GDM about the best way to feed their babies. Some women expressed a fear of breastfeeding as it could pass diabetes through to the child.

"*“If the mother breastfeeds her baby the ‘diabetes factor’ may transmit to the baby and it’s no good. It may make the baby have the same disease afterwards”*"

Other women were aware of health benefits associated with breast-feeding and stated that despite the perceived risk of transmission, they would still prefer to breast-feed:

"*“I’m concerned about that (transmission of diabetes to the infant) of course. However to my understanding breast milk has antibodies which might be good for my baby because they would protect it from infection”*"

### The desire for more information about GDM

The majority of women who participated in the focus groups expressed a strong desire to obtain more information. Women felt it would be particularly helpful to have more detailed information on the recommended dietary modifications and the risks of GDM for the baby. They felt the doctors provided useful information, however were too busy to answer all their questions:

"*“…Doctors have to consult for many patients. In fact they may have so much work to do and this disease lasts for quite a long time during your pregnancy, so you may sometimes (have) lack of consultation”*"

"*“We have many questions but dare not keep asking”*"

"*“There is just a small sheet concerning the risk of GDM distributed by the hospital staff at the antenatal care department. No other information on GDM is available”*"

When they could not get sufficient information from doctors, the women stated they found other sources of information.

"*“We read magazines, books or ask friends.”*"

"*“I asked other patients”*"

Another source of information used was a phone health support service, which is run by non-medical personnel and is not specifically for diabetes or pregnancy. Other participants stated they visited Government Nutrition centres, which have been established to prevent childhood under nutrition.

When women were asked about how they would like to receive information on GDM,women felt that facilitated small group sessions with medical practitioners as well as more detailed leaflets specifically describing dietary changes would be useful.

## Discussion

To our knowledge this is the first qualitative study describing the attitudes and health behaviours of women with GDM in Vietnam. We believe it has relevance for clinicians in Vietnam and other low and middle-income settings managing women with GDM. We demonstrate a lack of health literacy and knowledge of GDM in Vietnamese women, in the context of a desire by the women to have more information made available to them. Lack of knowledge resulted in women with GDM feeling anxiety and concern about their diagnosis. Several misconceptions about the effects of GDM, dietary changes and breastfeeding were identified through the focus group process, and this information will be important in designing future health promotion programs in this setting.

The strengths of the study are the purposive sampling in order to obtain a representation of views of women with differing severities of GDM. A limitation is that this is a single centre study, and experiences of pregnant women at other centres may differ depending on the resources available for counselling and management. Whilst this study involved only small number of women, the major themes were expressed in all groups, which strengthens the internal validity.

During the process of analysing data, we remained aware of the effects of the prior experience of the facilitator and researchers that would influence the interpretation of findings (reflexivity). The status of the facilitator (TST) as a respected male doctor was acknowledged as a potential source of bias. He was not involved directly in the clinical care of any of the participants and questions were carefully structured to minimise facilitator preconceptions.

Other studies that have examined the attitudes of women with GDM in pregnancy all come from high-income country contexts [20-28] (Table3). 

**Table 3:** Summary of publications from 1989-2012 describing attitudes of women with GDM

	**Location/Study population/controls**	**Total n**	**Study methodology**	**Main findings**
Spirito 1989 [20]	Rhode Island, USA. Diabetes in Pregnancy Program. 68 GDM and 50 controls	118	45 min semi structured interview and self completed Profile of Mood States-Bipolar Form	No significant difference in emotional state of women with GDM or controls.
Lawson, 1994 [21]	Recently diagnosed GDM in Kentucky, USA	17	Interviews: once prenatally and once post partum	Most women experienced fear, anxiety and depression following diagnosis. Diet posed multiple difficulties and challenges. Respondents reported experimenting with different foods and primary concern being fetal wellbeing.
Sjogren 1994 [22]	Sweden. 113 post partum women who had GDM and 226 controls	339	Self completed questionnaire after pregnancy	Women with GDM reported poorer well-being, increased worry, decreased psychic health and energy. No difference in breast-feeding rates between groups.
Langer 1994 [23]	San Antionio, Tx USA. 206 GDM and 95 controls in a low socioeconomic area.	301	Self administered Profile of Mood States- Bipolar Form	GDM does not adversely contribute to emotional state.
Rumbold 2002 [24]	Adelaide Australia. Women surveyed prior to being screened for GDM, after the screening test when results were known and around 36 weeks gestation. 25 GDM, 184 controls	209	Self-administered questionnaire Spielberger State-Trait anxiety inventory, Edinburgh Post Natal Depression score and SF-36 health status.	No differences in anxiety or depression scores were found after screening or later in pregnancy between those screening positive or negative. Women screening negative had better health perceptions than those screening positive, however differences not evident later in pregnancy.
Hjelm 2005 [25]	Specialist diabetes in pregnancy clinic in Lund Sweden, 13 women born in Sweden and 14 in Middle East	27	Semi structured interviews	Negative feelings and worries when informed about diagnosis. All women concerned about health of baby. Women from Middle East knew less about causes and consequences of GDM
Evans 2005 [26]	Diabetic outpatient clinic, Western Canada	12	In depth interviews, twice in pregnancy and once 6-8 weeks postpartum.	Main theme of ‘living a controlled pregnancy’. Women acknowledged their role in controlling their diabetes for the sake of their baby, but found it very difficult at times. Although they recognised the adverse effects, many perceived the experience as beneficial. Their knowledge about pregnancy, body and diet perceived as empowering.
Hjelm, 2008 [27]	A specialist diabetes clinic and specialist midwifery clinic in Lund, Sweden	23	Semi structured interviews	Negative feelings around diagnosis. Recommended that more information be provided immediately after diagnosis of GDM and continually reinforced.
Daniells 2003 [28]	Wollongong, Australia.56 women with GDM and 50 controls.	106	Self-completed questionnaire Mental Health Inventory (MHI-5) and Speilberger state-trait anxiety inventory administered at diagnosis, 36 weeks and 6 weeks post partum.	Few differences in mental health and anxiety measures between the groups. At time of diagnosis GDM women reported significantly greater psychological distress on the MHI-5 and state anxiety scores. These scores similar by 36 weeks and postpartum.
Collier 2011 [17]	Atlanta, USA. Women who had had a pregnancy affected by GDM (54 women) or pre-existing DM (35 women) in the last 4 years	89	Focus groups	Barriers to management of GDM included financial, access to care, barriers to physical activity and diet, information barriers. Participants reported feeling alone and overwhelmed by diabetes requirements. Lack of knowledge in GDM group about effects on long term health
Bandyopadhyay 2011 [18]	Melbourne, Australia. 17 immigrant women from South Asia	17	In depth interviews	Fear, shock and distress with diagnosis. Difficulties adapting to dietary advice as too general. Concern that restricted diet would affect baby’s growth. Restriction of key traditional foods. Paramount concerns for the baby. Fear of injecting insulin

They demonstrate several parallels, particularly in women who are from migrant groups and low socioeconomic settings. Yet there were some notable differences reported in our study by the Vietnamese women, which most likely relate to the lack of resources available in this setting.

### Anxiety about the diagnosis of GDM

Vietnamese women felt worried and anxious about their diagnosis of GDM. Maternal anxiety associated with GDM has been examined in studies from Australia [24,28], the USA [17], Canada [26] and Sweden [22]. Whilst there was variation in the methods of assessment used and the external validity of each study, overall they reported that mothers seemed initially anxious when told they had GDM however this anxiety decreased as the pregnancy progressed. The Australian Carbohydrate Intolerance Study in Pregnant Women (ACHOIS) randomised control trial [8], conducted in Australia, measured maternal health status using the SF-36 question scale. The authors demonstrated a trend towards improved perception of health in the group of women treated for GDM. These studies were all conducted in the setting of comprehensive multidisciplinary care. This lends support for increasing the information available to women and their contact with health providers as a means to potentially reduce maternal anxiety around GDM in Vietnam.

### Dietary confusion

Vietnamese women in our study stated that they felt hunger and were concerned that the diet was insufficient for adequate fetal growth. These same feelings were identified in immigrant women from South Asia living in Melbourne, Australia [18]. These women felt that key elements of their traditional diet were restricted. The Vietnamese women expressed similar frustrations, with the reported instruction to reduce starch interpreted as reducing white rice, the dietary staple. They expressed confusion about how to replace the calories white rice provided from other food sources, with a fear of high BGL leading to severe dietary restriction in some cases. Other women reported experimenting with different foods as they lacked specific instructions on what to eat. There are no culturally specific dietary guidelines for women who develop GDM in Vietnam, although these have been developed for expatriate Vietnamese women in Australia [29]. These guidelines could potentially be adapted for use in Vietnam in conjunction with health professionals and consumer groups.

### Blood glucose monitoring

The findings from this study highlight that despite a cost-neutral loan scheme for blood glucose meters, the majority of women still came to the hospital for monitoring. There is a lack of high quality evidence to guide the recommendations for the frequency of BGL monitoring, however the regular monitoring of fasting and post-prandial glucose levels has been associated with a reduction in rates of neonatal macrosomia [30]. For women with mild, diet controlled GDM; there is some evidence that weekly fasting glucose levels may be adequate [31], although there are no studies supporting this from this setting. It was concerning that some women receiving insulin injections were monitoring BGL once a week or less. This exposes women to the potential for hypoglycaemic episodes or sub-adequate glycaemic control resulting in perinatal complications. To date, metformin and other oral hypoglycaemic agents are not licensed for use in pregnancy in Vietnam. Given the encouraging evidence of the safety and efficacy of metformin in the management of women with GDM [32], it is possible that this could provide a safer method of glycaemic control for women in Vietnam and other low resource settings in the future.

### Breast feeding

Several women expressed a preference not to breastfeed for fear of transmission of the “diabetes factor” to the baby. Only a minority of women expressed a desire to breastfeed. This contrasts to the UK where 95.5% of mothers with GDM in one study (n = 68) stated during pregnancy that they intended to breastfeed [33]. Vietnam is one of the countries with the lowest rates of exclusive breast-feeding among infants less than 4 or 6 months old [34]. In a review of infant feeding practices and barriers to breast-feeding in Vietnam, Nguyen et al in 2011 identified the lack of lactation support staff in hospitals and the aggressive marketing of formula milk within maternity hospitals as contributory [35]. Health care providers need to be educated to include information on the importance of breast-feeding when counselling women with GDM. Breast-feeding is one of the few interventions that can reduce the risk childhood and adolescent obesity in the offspring of these women [36].

### Improving access to information

What this study has demonstrated most conclusively is that women in Vietnam lack the information to enable them to prevent adverse outcomes from GDM. In the USA, Collier et al in 2011 identified that the major concerns amongst low socio-economic status women with GDM were the lack of information about GDM and barriers to receiving and accessing health care [17]. Women in our study stated there was a lack of written information available on GDM, and that they felt health providers lacked sufficient time for counselling.

We did not investigate the views and knowledge of the health care providers. In addition to improving patient access to resources, it will be essential to address clinician knowledge if improvements in health literacy are to be made in this setting.

## Conclusion

This qualitative study gives novel insights into the way in which a diagnosis of GDM effects women in a low-middle income country context. It is particularly timely given the acknowledgement of the growing global burden of non-communicable diseases at the 2011 United Nations High Level Meeting [37] and the need to develop patient centred care models in all settings. Internationally there is much debate around the best method to screen for GDM. This study illustrates that screening for diabetes is just the first step in preventing adverse perinatal outcomes. Without adequate education and support for women and health care providers it is possible that screening could worsen rather than improve perinatal and psychological outcomes. We recommend that in Vietnam, the scale up of screening for GDM needs to be accompanied by a comprehensive clinician education and patient health promotion package for pregnancy, the postnatal period and beyond. Culturally specific advice on diet and the promotion of breast-feeding in women with GDM should be the first steps in ensuring the optimal health of these women and their offspring.

## Abbreviations

GDM, Gestational Diabetes Mellitus.

## Competing interests

None of the authors have any financial or non-financial competing interests to declare.

## Authors’ contributions

JEH devised the study, performed the analysis and drafted the manuscript. TST devised the study, performed the focus groups, edited the transcripts and edited the manuscript. MAD translated and transcribed the transcripts and edited the manuscript. RF provided advice on qualitative research methods and analysis and edited the manuscript. JMM devised the study and edited the manuscript. HEJ devised the study and edited the manuscript. All authors read and approved the final manuscript.

## Pre-publication history

The pre-publication history for this paper can be accessed here:

http://www.biomedcentral.com/1471-2393/12/81/prepub

## References

[B1] IDF Diabetes Atlas 2011 5 International Diabetes Federation, Brussels, Belguim Accessed 19 Arpil 2012: http://www.idf.org/diabetesatlas 35914061

[B2] Metzger BE Coustan DR Summary and recommendations of the Fourth International Workshop-Conference on Gestational Diabetes Mellitus Diabetes Care 1998 21 Suppl 2 B161 B167 9704245

[B3] Zhang F Dong L Zhang CP Li B Wen J Gao W Increasing prevalence of gestational diabetes mellitus in Chinese women from 1999 to 2008 Diabet Med 2011 28 6 652 657 10.1111/j.1464-5491.2010.03205.x 21569085

[B4] Seshiah V Balaji V Balaji MS Paneerselvam A Kapur A Pregnancy and diabetes scenario around the world: India Int J Gynaecol Obstet 2009 104 Suppl 1 S35 S38 19154999 10.1016/j.ijgo.2008.11.035

[B5] Chan J Malik V Jia W Kadowaki T Yajnik C Yoon K Diabetes in Asia epidemiology, risk factors and pathophysiology JAMA 2009 301 2129 2141 10.1001/jama.2009.726 19470990

[B6] Ben-Haroush A Yogev Y Hod M Epidemiology of gestational diabetes mellitus and its association with Type 2 diabetes Diabet Med 2004 21 2 103 113 10.1046/j.1464-5491.2003.00985.x 14984444

[B7] IDF Diabetes in Pregnancy: Protecting Maternal Health 2012 International Diabetes Federation Policy Briefing, Accessed 20 April 2012 [http://www.idf.org/node/2298]

[B8] Crowther CA Hiller JE Moss JR McPhee AJ Jeffries WS Robinson JS Effect of treatment of gestational diabetes mellitus on pregnancy outcomes N Engl J Med 2005 352 24 2477 2486 10.1056/NEJMoa042973 15951574

[B9] Landon MB Spong CY Thom E Carpenter MW Ramin SM Casey B A multicenter, randomized trial of treatment for mild gestational diabetes N Engl J Med 2009 361 14 1339 1348 10.1056/NEJMoa0902430 19797280 PMC2804874

[B10] Tieu J Middleton P McPhee AJ Crowther CA Screening and subsequent management for gestational diabetes for improving maternal and infant health Cochrane Database Syst Rev 2010 7 CD007222 20614455 10.1002/14651858.CD007222.pub2 PMC4161118

[B11] Kim C Newton K Knopp R Gestational diabetes and the incidence of type 2 diabetes: a systematic review Diabetes Care 2002 25 1862 1868 10.2337/diacare.25.10.1862 12351492

[B12] American Diabetes Association Diagnosis and classification of diabetes mellitus Diabetes Care 2010 33 Suppl 1 S62 S69 20042775 10.2337/dc10-S062 PMC2797383

[B13] Metzger BE Gabbe SG Persson B Buchanan TA Catalano PA Damm P International association of diabetes and pregnancy study groups recommendations on the diagnosis and classification of hyperglycemia in pregnancy Diabetes Care 2010 33 3 676 682 20190296 10.2337/dc09-1848 PMC2827530

[B14] Hirst JE Tran TS Do MAT Morris JM Jeffery HE Consequences of gestational diabetes in an urban hospital in Vietnam: a prospective cohort study PLoS Med 2012 9 7 e1001272 10.1371/journal.pmed.1001272 22911157 PMC3404117

[B15] Lawrence JM Women with diabetes in pregnancy: different perceptions and expectations Best Pract Res Clin Obstet Gynaecol 2011 25 1 15 24 10.1016/j.bpobgyn.2010.10.003 21115403

[B16] Rosenstock IM Strecher VJ Becker MH Social learning theory and the Health Belief Model Health Educ Q 1988 15 2 175 183 10.1177/109019818801500203 3378902

[B17] Collier SA Mulholland C Williams J Mersereau P Turay K A qualitative study of perceived barriers to management of diabetes among women with a history of diabetes during pregnancy J Womens Health (Larchmt) 2011 20 9 1333 1339 10.1089/jwh.2010.2676 21740191

[B18] Bandyopadhyay M Small R Davey MA Oats JJ Forster DA Aylward A Lived experience of gestational diabetes mellitus among immigrant South Asian women in Australia Aust N Z J Obstet Gynaecol 2011 51 4 360 364 10.1111/j.1479-828X.2011.01322.x 21806582

[B19] Pope C Campbell R Qualitative research in obstetrics and gynaecology BJOG 2001 108 3 233 237 11281460 10.1111/j.1471-0528.2001.00077.x

[B20] Spirito A Williams C Ruggiero L Bond A McGarvey ST Coustan D Psychological impact of the diagnosis of gestational diabetes Obstet Gynecol 1989 73 4 562 566 2648224

[B21] Lawson E Rajaram S A transformed pregnancy: the psychosocial consequences of gestational diabetes Sociol Health Illn 1994 16 4 536 562 10.1111/1467-9566.ep11347644

[B22] Sjogren B Robeus N Hansson U Gestational diabetes: a case-control study of women's experience of pregnancy, health and the child Journal of Psychosom Res 1994 38 8 815 822 10.1016/0022-3999(94)90069-8 7722961

[B23] Langer N Langer O Emotional adjustment to diagnosis and intensified treatment of gestational diabetes Obstet Gynecol 1994 84 3 329 334 8058225

[B24] Rumbold AR Crowther CA Women's experiences of being screened for gestational diabetes mellitus Aust N Z J Obstet Gynaecol 2002 42 2 131 137 10.1111/j.0004-8666.2002.00131.x 12069138

[B25] Hjelm K Bard K Nyberg P Apelqvist J Swedish and Middle-Eastern-born women's beliefs about gestational diabetes Midwifery 2005 21 1 44 60 10.1016/j.midw.2004.09.004 15740816

[B26] Evans MK O'Brien B Gestational diabetes: the meaning of an at-risk pregnancy Qual Health Res 2005 15 1 66 81 10.1177/1049732304270825 15574716

[B27] Hjelm K Berntorp K Frid A Aberg A Apelqvist J Beliefs about health and illness in women managed for gestational diabetes in two organisations Midwifery 2008 24 2 168 182 10.1016/j.midw.2006.12.008 17360084

[B28] Daniells S Grenyer BF Davis WS Coleman KJ Burgess JA Moses RG Gestational diabetes mellitus: is a diagnosis associated with an increase in maternal anxiety and stress in the short and intermediate term? Diabetes Care 2003 26 2 385 389 10.2337/diacare.26.2.385 12547867

[B29] Cultural meal guide for Vietnamese women with gestational diabetes AHS-6715 2009 Fairfield Hospital, NSW Australia Accesed 1 May 2012. [http://www.mhcs.health.nsw.gov.au/topics/diabetes.html]

[B30] Hawkins JS Glucose monitoring during pregnancy Curr Diab Rep 2010 10 3 229 234 10.1007/s11892-010-0111-9 20425587 PMC2856850

[B31] Homko CJ Sivan E Reece EA The impact of self-monitoring of blood glucose on self-efficacy and pregnancy outcomes in women with diet-controlled gestational diabetes Diabetes Educ 2002 28 3 435 443 10.1177/014572170202800313 12073958

[B32] Rowan JA Hague WM Gao W Battin MR Moore MP Metformin versus insulin for the treatment of gestational diabetes N Engl J Med 2008 358 19 2003 2015 10.1056/NEJMoa0707193 18463376

[B33] Soltani H Arden M Factors associated with breastfeeding up to 6 months postpartum in mothers with diabetes Journal of Obstetric, Gynecologic, and Neonatal Nursing : JOGNN / NAACOG 2009 38 5 586 594 10.1111/j.1552-6909.2009.01052.x 19883480

[B34] UNICEF Childinfo Monitoring the situation of women and children 2002, Accessed 15 April 2012 [http://www.childinfo.org/breastfeeding_vietnam.html]

[B35] Nguyen PH Menon P Ruel M Hajeebhoy N A situational review of infant and young child feeding practices and interventions in Viet Nam Asia Pac J Clin Nutr 2011 20 3 359 374 21859654

[B36] Schaefer-Graf UM Hartmann R Pawliczak J Passow D Abou-Dakn M Vetter K Association of breast-feeding and early childhood overweight in children from mothers with gestational diabetes mellitus Diabetes Care 2006 29 5 1105 1107 10.2337/dc05-2413 16644645

[B37] Beaglehole R Bonita R Alleyne G Horton R Li L Lincoln P UN High-Level Meeting on Non-Communicable Diseases: addressing four questions Lancet 2011 378 9789 449 455 10.1016/S0140-6736(11)60879-9 21665266

